# Chicago Medical Society

**Published:** 1879-12

**Authors:** 


### ARTICLE IX.

CHICAGO MEDICAL SOCIETY. Meeting Nov. 3d, 1879. Dr. ANDREWS, President, in the chair.

Dr. L. H. MONTGOMERY reported a case of spina bifida and hydrocephalus, which had some interesting points.

The child was born Aug. 6th, and died Nov. 2nd, 1879. The spinal tumor was about the size of an orange, was in the lumbar region, and covered with a superficial ulcer, was indistinctly lobulated, and opened externally by a fistulous tract. At first it was quite elastic, especially with the child in an upright position; but when the child was laid in a horizontal position it was soft and flabby, becoming tense, however, when the child cried. There was no paraplegia for some weeks after birth. After the superficial ulceration had healed, under appropriate treatment, and the fistula closed, it was noticed that the head began to enlarge. Compression of the tumor below, caused the cranial walls to bulge, and pressure on the vertex caused the tumor to distend For some time the child appeared to thrive, but after a time the hydrocephalus became more and more marked, and the child finally succumbed to asthenia, dying at the eighty-fifth day.

In the discussion which followed, it appeared to be the general opinion that tapping and external pressure yielded the best results in the treatment of spina bifida.

---

MEETING NOV. 17th. Dr. ANDREWS in the chair.

Drs. FENGER and SALISBURY presented a communication on fatty embolism of the brain and lungs, following fracture. They reported a case which followed a fracture of the femur in a middle-aged woman, and Dr. Fenger exhibited a number of sections under the microscope, giving evidence of the lesion. The paper gave a general résumé of the subject, and will appear in the JOURNAL AND EXAMINER.

Dr. R. PARK related a case of sudden coma, with fatal termination, a comparatively short time after operation for caries of the tarsus, which he thought was most rationally accounted for on supposition that there had been a fatty infarction in the lungs, with consequent anæmia of the brain, the symptoms corresponding well with those described in the paper.

---

CATGUT DRAINAGE. By Jules Boeckel. (*Revue Méd. de l'Est*, June 1, 1879.)

Dr. Jules Boeckel, having used the catgut drainage in a number of capital operations, insists that it can be employed with advantage over the ordinary rubber drainage tube. He used nine catgut threads, the ninth being wound round the eight to keep them together, the two ends being fastened together by a piece of silk. In every case reported, the catgut used as drainage became absorbed after about the sixth day. The catgut, he claims, affords sufficient drainage; it does not act as a foreign body as it is readily absorbed; it does not require the attention which the ordinary rubber tube does; and the separation of the tissues is reduced to a minimum. Theoretically, he says one single catgut thread should be sufficient. Another advantage claimed for it is that compression can be made to any extent so as to bring the deeper parts in close apposition without fear of obstructing the drainage.

WE join the *Southern Clinic* and the *Louisville Medical News* in asking the question, Who is or was Dr. F. A. Ticknor, of Columbus, the author of "Little Giffen, of Tennessee." The poem is worthy to be ranked with some of those from the pen of another member of the profession, Dr. O. W. Holmes.

